# Increased serum concentration of netrin-1 after intravitreal bevacizumab injection: is it a compensatory mechanism to counteract drug side effects?

**DOI:** 10.1186/s12886-021-01989-1

**Published:** 2021-05-31

**Authors:** Murat Okutucu, Hüseyin Fındık, Mehmet Gökhan Aslan, Medeni Arpa

**Affiliations:** Recep Tayyip Erdoğan University, Zihni Derin Yerleşkesi - Fener Mahallesi, 53100 Merkez/Rize, Turkey

**Keywords:** Vascular endothelial growth factor, Netrin-1, Bevacizumab, Macular edema, Diabetic retinopathy

## Abstract

**Background:**

To evaluate alterations in the serum concentrations of vascular endothelial growth factor (VEGF) and netrin-1 after intravitreal bevacizumab (BCZ) injection for the treatment of diabetic macular edema (DME).

**Methods:**

This prospective case-control study included a total of 50 participants assigned to one of three groups, including 10 individuals with DME and non-proliferative diabetic retinopathy (NPDR), 13 with DME, and proliferative diabetic retinopathy (PDR), and 27 healthy individuals as a control group. Serum VEGF and netrin-1 concentrations were measured by enzyme-linked immunosorbent assays (ELISAs) immediately before, as well as 1 week and 1 month after, intravitreal BCZ injection.

**Results:**

The mean VEGF serum concentrations in the PDR and NPDR groups were 388.4 and 196.9 pg/mL at baseline, respectively. After 1 week, these concentrations changed to 193.41 and 150.23 pg/mL, respectively (*P* = 0.001 and *P* = 0.005, respectively); after 1 month, the concentrations were 97.89 and 76.46 pg/mL, respectively (*P* = 0.001 and *P* = 0.009, respectively). The mean netrin-1 serum concentrations in the PDR patients and NPDR groups were 318.2 and 252.7 pg/mL at baseline, respectively. After 1 week, these concentrations increased to 476.6 and 416.3 pg/mL, respectively (*P* = 0.033 and *P* = 0.005, respectively), and after 1 month, they were 676.6 and 747.5 pg/mL, respectively (*P* = 0.001 and *P* = 0.005, respectively). The correlation analysis revealed a significant inverse relationship between changes in serum VEGF and netrin-1 concentrations in both the PDR and NPDR groups (r = − 0.685, *P* = 0.029).

**Conclusions:**

Intravitreal BCZ injections work systemically to significantly decrease serum VEGF levels, leading to a significant upregulation in the concentration of another angiogenic mediator, netrin-1.

## Background

Diabetic macular edema (DME) occurs as a result of fluid leakage from abnormal perifoveal retinal capillaries or microaneurysms into the intraretinal and subretinal areas. The pathogenesis of DME is multifactorial, and it is accompanied by the destruction of both the blood-retinal barrier (BRB) and the blood-aqueous barrier. Several studies have detected elevated vascular endothelial growth factor (VEGF) and pro-inflammatory cytokine concentrations in the aqueous humor of DME patients, as well as an increase in the concentrations of netrin-1 [1, 2].

VEGF signaling contributes to DME by increasing the vasopermeability of both intraretinal and subretinal vessels [3]. Netrin-1 can both enhance the pro-angiogenic function of VEGF and regulate the adhesion of vascular smooth muscle cells and endothelial cells by binding to neogenin [4]. Yu et al. detected increased BRB breakdown in diabetic rats after intravitreal (0.1 μg/mL) netrin-1 injection [5].

Tu et al. reported that netrin 1 binds with high affinity to CD146 on the vascular endothelium and that the netrin-1-CD146 interaction is necessary for downstream VEGF signal transduction and endothelial cell activation induced by netrin-1. In addition, CD146 acts as a co-receptor for VEGFR2 to facilitate the transduction of VEGF signaling in endothelial cells [6].

Bevacizumab (BCZ) (Avastin, Genentech, Inc., San Francisco, California, USA) is an anti-VEGF drug used off-label to treat DME [7, 8]. There have been few studies in the literature that have evaluated plasma VEGF levels after intravitreal BCZ injection; however, none of these evaluated serum concentrations of netrin-1, which has similar VEGF-like physiological activity [9–11]. Therefore, we aimed to evaluate changes in the serum concentrations of both netrin-1 and VEGF after intravitreal BCZ injection to better understanding drug side effects.

## Methods

### Study participants

This observational case-control study was conducted between April 2019 and February 2020 in the ophthalmology clinic of Recep Tayyip Erdoğan Training and Research Hospital in affiliation with Recep Tayyip Erdoğan University. The study protocol was approved by the Recep Tayyip Erdogan University Ethics Committee (Approval ID: 2019/48). Throughout the study, the tenets of the Declaration of Helsinki were followed. Written informed consent was obtained from all subjects prior to participation. The study was comprised of three groups, with a total of 50 participants; these included 10 DME patients with non-proliferative diabetic retinopathy (NPDR) in the NPDR group, 13 DME patients with proliferative diabetic retinopathy (PDR) in the PDR group, and 27 age-sex matched non-diabetic individuals acting as the control group. Patients were diagnosed with PDR, NPDR, and DME according to the international clinical DR and DME disease severity scales criteria [12]. The exclusion criteria included having a history of diseases that may alter serum netrin-1 and VEGF concentrations, such as age-related macular degeneration (AMD), retinal vein occlusion, glaucoma, cardiovascular disease, cerebrovascular and neurodegenerative diseases, collagen diseases (systemic lupus erythematosus, polymyositis/dermatomyositis), chronic inflammatory diseases (rheumatoid arthritis, ulcerative colitis, and Crohn’s disease), hematological diseases, malignancies, renal and hepatic diseases, and vasculitis. In addition, patients who had been receiving retinal photocoagulation treatment for at least 6 months before anti-VEGF injection, as well as those being treated with hydroxymethylglutaryl-coenzyme A (HMG-CoA) reductase inhibitors, corticosteroids, non-steroidal anti-inflammatory drugs (NSAIDs), including acetylsalicylic acid, and those using tobacco and/or alcohol were also excluded from the study.

The experimental groups consisted of type 2 diabetes mellitus patients who were over 40 years of age. Patients with vision loss due to DME and a macular thickness ≥ 250 μm at the 1 mm diameter of the central fovea were included in the study. Patients with accompanying hypertensive diseases were also included if their blood pressure was under control based on measurements taken before and after injections. Individuals who were planned to receive an elective phacoemulsification cataract surgery with no history of any systemic disease other than hypertension and who were over 40 years of age constituted the control group. All patients underwent detailed ophthalmic examinations, including assessment of corrected distance visual acuity (CDVA), slit-lamp examination, tonometry, gonioscopy, dilated fundus examination, and optic coherence tomography (OCT).

### Visual acuity and cube average thickness (CAT) assessment

The CDVA was evaluated by a Snellen letter chart and converted to a logarithm of the minimum angle of resolution (logMAR) vision score for statistical analysis. Macular thickness alterations (including central subfield thickness, CAT, and cube volume) were determined using a Cirrus *high-definition* spectral-domain (HD SD) OCT device (Carl Zeiss Meditec, Dublin, CA, USA). The scan pattern was based on a 6 mm × 6 mm data cube captured by macular cube scans (200 × 200 mode). The Early Treatment Diabetic Retinopathy Study (ETDRS) grid was automatically centered on the fovea with a fovea finder. The retinal thickness value, from the internal limiting membrane to the retinal pigment epithelium, was measured in microns. We used only the CAT values for statistical analysis, and DME was diagnosed as a thickness ≥ 250 μm at the 1 mm diameter of the central fovea.

### Collection of blood samples

Venous blood samples (5 mL) were collected from the participants in the experimental groups immediately before and 1 week and 1 month after BCZ injection; the samples were kept in serum-separating tubes. Blood samples were collected from the control group participants prior to cataract surgery. The coagulated blood samples were centrifuged at 3000×g for 10 min to obtain the sera, which were stored at − 20 °C until the quantification of VEGF and netrin-1 concentrations was performed.

### Intravitreal injections

Intravitreal BCZ injection was performed in the operating room under sterile conditions. BCZ (Avastin 100 mg/4 mL; 1.25 mg in a 0.05 mL volume) was injected into the vitreous humor with a 30-gauge sharp-tipped needle at a depth of 4 mm behind the limbus, after which the needle was carefully removed. A sterile cotton applicator was used to prevent reflux. Topical antibiotics were applied four times a day for 1 week postoperatively. Intraocular pressure remained stable in all patients in postoperative examinations and none needed anti-glaucomatous medications.

### Quantification of netrin-1 and VEGF concentrations

Serum netrin-1 concentrations were determined by enzyme-linked immunosorbent assay (ELISA) kits (SunRed Bio, Shanghai, China, Catalogue No: 201–12-1278) according to the instructions of the manufacturer. Serum VEGF concentrations were determined by different ELISA kits (Elabscience, Houston, USA, Catalogue No: E-EL-H011196T), as suggested by the manufacturer.

### Sample size

A sample size calculation was performed with the G-power software (v3.1.9.2) program (G-power v3.1.9.2, Universitat Kiel, Kiel, Germany). The total sample size was calculated as 40 participants in 3 groups for 3 repeated measurements to obtain 95% power, with 0.05 α-error and 0.5 effect size.

### Data analysis

Statistical analyses were performed using Statistical Package for the Social Sciences (SPSS) version 23.0 for Windows software (SPSS, Inc., Chicago, IL, USA). The distributions of the variables were investigated using Shapiro-Wilk tests to assess normality. Descriptive statistics are presented as means ± SDs for normally distributed variables and as medians with minimum and maximum values for non-parametric variables. One-way analysis of variance (ANOVA) and Kruskal –Wallis tests were used to compare parametric and non-parametric variables, respectively, among the groups. Bonferroni-corrected Mann-Whitney U tests were used for comparing non-normally distributed data between subgroups. Independent samples t-tests were used for comparing normally distributed data between groups. Friedman tests were used for the repeated-measures analysis of variance by rank comparisons of three or more groups (at baseline, as well as 1 week and 1 month after BCZ injection). Wilcoxon signed-rank tests were used to compare non-parametric variables between time points at baseline and the end of the first month. Spearman’s correlation coefficients were used to identify correlations between serum concentrations of VEGF and DRP severity or CAT values. Chi-square tests were used to compare categorical variables. The level of statistical significance was set at *p* < 0.05.

## Results

The baseline demographic, clinical, and ocular characteristics of the participants are presented in Table 1. Although there were no significant differences among the three groups in terms of age, sex, and level of hypertension (HT), there was a significant difference between the groups in terms of baseline serum concentrations of netrin-1 and VEGF (Tables 1 and 2). Baseline LogMAR and CAT values showed no significant differences between the NPDR and PDR groups. (*p* = 0.648, *p* = 0.208, respectively). The baseline serum netrin-1 levels were significantly higher in the control group compared to both NPDR and PDR groups (all Ps < 0.001); however, there was no significant difference between the NPDR and PDR groups (*P* = 0.115) (Table 2). The baseline VEGF concentration was higher in the PDR group compared to the NPDR and control groups (*P* = 0.002 and *P* < 0.001, respectively) (Table 2). In both DME groups (NPDR and PDR), there was no significant relationship between serum netrin-1 concentrations, logMAR, and CAT levels (r = − 0.075, *P* = 0.735, and r = 0.211, *P* = 0.334, respectively) and VEGF concentrations, logMAR, and CAT levels (r = − 0.104, *P* = 0.636, and r = 0.182, *P* = 0.405, respectively) before intraocular injection.

**Table 1:** Demographic and clinical data of the study groups

	Control group (***n*** = 27)	NPDR group (***n*** = 10)	PDR group (***n*** = 13)	***P***-value
Hypertension, n (HT+/HT-)	11/16	4/6	6/7	0.939^a^
Sex, n (F/M)	13/14	3/7	7/6	0.496^a^
Age (years) (mean ± SD)	65.74 ± 7.17	66.70 ± 12.37	67.85 ± 8.93	0.778^b^

*F* Female, *M* Male, *n* Count, *HT(+)* Hypertension positive group, *HT( –)* Hypertension negative group, *SD* Standard deviation Note: ^a^Chi-Square test, ^b^One Way ANOVA test

**Table 2:** Baseline ocular characteristics of the patients in each group

		Control group	NPDR group	PDR group	***P***-value
Baseline netrin-1 (pg/ml)	mean ± SD	983 ± 664	253 ± 75	318 ± 68	
	median (IQR)	737 (597–931)	245 (216–321)	301 (272–323)	< 0.001^a^
Baseline VEGF (pg/ml)	mean ± SD	203 ± 137	197 ± 78	388 ± 232	
	median (IQR)	157 (122–263)	184 (155–217)	320 (288–377)	0.001^a^

*NPDR* Non-proliferative diabetic retinopathy, *PDR* Proliferative diabetic retinopathy, *IQR* Interquartile range, *SD* Standard deviation, *VEGF* Vascular endothelial growth factor. Note: ^a^Kruskal-Wallis Test

The logMAR and CAT values significantly improved in the NPDR group 1 month after the BCZ injection compared to the pre-injection baseline values (*P* = 0.011 and *P* = 0.005, respectively). The logMAR and CAT values significantly improved in the PDR group 1 month after the BCZ injection compared to the pre-injection baseline values (all Ps = 0.001, 0.001) (Table 3). However, the improvements in logMAR and CAT values did not significantly differ between the two DME groups (*P* = 0.200 and *P* = 0.644, respectively).

**Table 3:** Intra-group comparison of vision and cube average thickness values before and 1 month after bevacizumab injection

	Baseline VA (LogMAR) median (IQR)	1 Month VA (LogMAR) median (IQR)	***P***-value	Baseline CAT (μm) median (IQR)	1 Month CAT (μm) median (IQR)	***P***-value
NPDR group	0.9 (0.5–1.0)	0.4 (0.2–0.7)	0.011^e^	337 (297–361)	301 (269–324)	0.005^a^
PDR group	0.8 (0.5–1.0)	0.3 (0.2–0.5)	0.001^e^	364 (341–378)	305 (288–337)	0.001^a^

*VA* Visual acuity, LogMAR The logarithm of the minimum resolution angle, *IQR* Interquartile range, *CAT* Cube average thickness, *NPDR* Non-proliferative diabetic retinopathy, *PDR* Proliferative diabetic retinopathy Note: ^a^Wilcoxon Rank Test

**Fig. 1:**
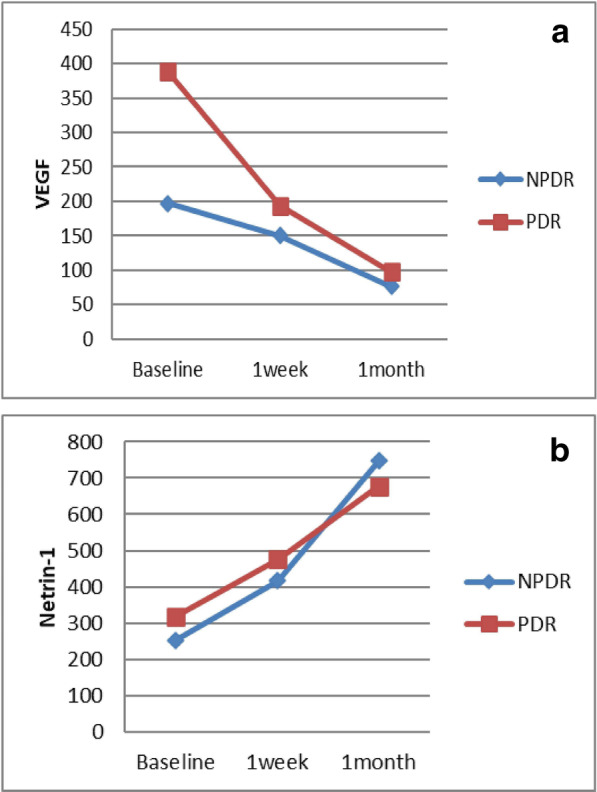
**a** Vascular endothelial growth factor (VEGF) concentrations at baseline, 1 week, and 1 month after bevacizumab (BCZ) injection in the non-proliferative diabetic retinopathy (NPDR) and proliferative diabetic retinopathy (PDR) groups. **b** Netrin-1 concentrations before and 1 week and 1 month after bevacizumab (BCZ) injection in the non-proliferative diabetic retinopathy (NPDR) and proliferative diabetic retinopathy (PDR) groups

The serum concentrations of VEGF significantly decreased in the NPDR group 1 week and 1 month post-injection compared to the pre-injection baseline values (*P* = 0.005 and *P* = 0.009, respectively) (Fig. 1A, Table 4). The serum concentrations of VEGF significantly decreased in the PDR group 1 week and 1 month post-injection compared to the pre-injection baseline values (all Ps = 0.001, respectively) (Fig. 1A, Table 4).

**Table 4:** Intra-group comparison of netrin-1 and vascular endothelial growth factor concentrations before and one week and one month after the injection of bevacizumab

		Baseline netrin-1 (pg/ml)	1 week netrin-1 (pg/ml)	1 month netrin-1 (pg/ml)	***P***-value	Baseline VEGF (pg/ml)	1 week VEGF (pg/ml)	1 month VEGF (pg/ml)	***P***-value
NPDR group	mean ± SD	252.7±74.6	416.3±123.0	747.5±89.6	< 0.0001^a^	196.9±78.2	150.23±96.23	76.46±53.91	< 0.002^a^
	median (IQR)	245,5 (213,8-324,8)	398,5 (317,3-456,6)	743,7 (689,7-827,6)		184,3 (155,6-221,8)	131,15 (87,40-187,50)	50,78 (41,40-98,50)	
PDR group	mean ± SD	318.2±68.4	476,6±225.0	676.6±187.5	< 0.0001^a^	388.4±232.4	193.41±89.13	97.89±53.08	< 0.0001^a^
	median (IQR)	300,5 (272,5-300,5)	377,0 (371,3-377,0)	719,7 (579,7-719,7)		319,5 (288,8-319,5)	161,20 (146,10-161,20)	83,34 (67,50-83,34)	

*VEGF* Vascular endothelial growth factor, *IQR* Interquartile range, *SD* Standard deviation, *NPDR* non-proliferative diabetic retinopathy, *PDR* Proliferative diabetic retinopathy

Note: ^a^Friedman test

The serum concentration of netrin-1 significantly increased in the NPDR group 1 week and 1 month post-injection compared to pre-injection baseline values (all Ps = 0.005) (Fig. 1B, Table 4). In the PDR group, a significant increase in netrin-1 concentration was only evident 1 month post-injection compared to baseline (*P* = 0.001) (Fig. 1B, Table 4).

The serum VEGF and netrin-1 concentrations, as well as the logMAR, and CAT values were examined to assess correlations in the DME groups. There was a significant moderate, negative relationship between the serum concentrations of VEGF and netrin-1 over the first month post-injection (r = − 0.685, *p* = 0.029) (Table 5).

**Table 5:** Correlation table showing the changes between vascular endothelial growth factor and netrin-1 concentrations, the logarithm of the minimum angle of resolution, and cube average thickness differences at one month post-bevacizumab injection in the non-proliferative diabetic retinopathy and proliferative diabetic retinopathy groups

				1 month netrin-1 difference	1 month VEGF difference	1 month VA (LogMAR) difference	1 month CAT (μm) difference
Spearman’s rho	NPDR Group	1 month netrin-1 difference	r	1.000	−0.685^a^	0.257	0.418
			p	.	0.029	0.474	0.229
			N	10	10	10	10
		1 month VEGF difference	r	−0.685^*^	1.000	−0.416	0.079
			p	0.029	.	0.232	0.829
			N	10	10	10	10
	PDR Group	1 month netrin-1 difference	r	1.000	−0.341	0.061	0.118
			p	.	0.255	0.842	0.700
			N	13	13	13	13
		1 month VEGF difference	r	−0.341	1.000	0.237	−0.066
			p	0.255	.	0.435	0.830
			N	13	13	13	13

^a^ Correlation is significant at the 0.05 level (2-tailed)

*VA* Visual acuity, *LogMAR* The logarithm of the minimum resolution angle, *CAT* Cube average thickness, *NPDR* Non-proliferative diabetic retinopathy, *PDR* Proliferative diabetic retinopathy, *VEGF* Vascular endothelial growth factor. r: Correlation Coefficient

## Discussion

In the current study, we observed that intravitreal injection of BCZ to treat DME resulted in a significant decrease in the serum concentration of VEGF and a significant increase in netrin-1, another angiogenic molecule. In the correlation analyses, we detected a significant moderate, negative relationship between reduced VEGF and increased netrin-1 concentrations.

Hirano et al. reported no correlation between plasma VEGF concentration and DME severity after the intravitreal injection of a single dose of BCZ, aflibercept, or ranibizumab (RBZ) for the treatment of DME. They also did not detect significant visual acuity improvements, despite the fact that plasma concentrations of VEGF were significantly decreased [9]. Consistent with that report, we also did not observe any correlation between serum VEGF and netrin-1 concentrations and DME severity (in the correlation analysis, CAT value, which is a more objective and numerical value, was used as an indicator of DME severity), despite observing a significant decrease in the amount of VEGF in serum. However, we did detect a significant increase in visual acuity 1 month after intravitreal BCZ injection. In the same study, Hirano et al. observed no differences between their NPDR, PDR, and control groups in terms of plasma concentrations of VEGF [9]. Even though we did not detect any difference in the baseline serum concentrations of VEGF between the NPDR and control groups, the concentration in the PDR group was significantly higher than that in the control group, consistent with the results reported by Wang et al. in PDR patients [13].

In the study of Hirano et al., two other points are inconsistent with the present study. First, according to the present study, serum VEGF level decreased significantly after the intravitreal bevacizumab injection in the first week and this decrease continued until the first month (in the other study, it reached its lowest level in the first week and increased again in the first month) [9]. Second, the VEGF levels that we quantitated were much higher. The reason for these inconsistencies is that the VEGF level was quantified in the plasma in the study of Hirano et al. [9] and serum in the present study. According to the recent study of Zou et al., the severity of retinopathy in diabetic patients is correlated with serum VEGF, not plasma VEGF, and serum VEGF levels are higher due to the VEGF released from platelets [14]. Besides, Verheul et al. showed that in patients receiving systemic bevacizumab treatment, bevacizumab was taken up by platelets, and inhibited VEGF in platelets [15]. Therefore, the VEGF level was quantified in serum in this study. Contrary to the other study, the reason serum VEGF level continued to decrease from the first week to the first month is that bevacizumab, which reaches the systemic circulation, continues to gradually decrease the stored VEGF in platelets.

Some of the previous studies have hypothesized that intravitreal BCZ injections suppress VEGF systemically and induce side effects due to the inhibition of VEGF-mediated physiological functions [9–11]; however, none of the previous studies focused on the response of other angiogenic mediators, such as netrin-1. Even though intravitreal injection of BCZ significantly decreased systemic VEGF concentrations in a previous study, the same effect was not observed after intravitreal RBZ injection [10]. Nevertheless, the efficacy and safety of both molecules are reported to be similar [16–19]. We detected significantly lower serum concentrations of netrin-1 in the NPDR and PDR groups compared to the control group, which was upregulated after intravitreal BCZ injection. The fact that the concentration was restored to normal physiological levels may explain the similar safety profiles of intravitreally administered RBZ and BCZ, and even though the passage of a small amount of BCZ into systemic circulation causes systemic VEGF suppression, a compensatory physiological response may be induced involving the upregulation of VEGF-like molecules such as netrin-1.

Kamba et al. conducted studies investigating the mechanisms of side effects resulting from systemic anti-VEGF therapy; they reported that systemic VEGF inhibition mostly caused side effects as a result of mechanisms related to decreased nitric oxide (NO) synthesis, destruction of the vascular endothelium, loss of endothelial fenestration of glomerular capillaries, proliferation of glomerular endothelial cells, loss of podocytes, inhibition of angiogenesis, and disruption of the blood-brain barrier [20]. Because netrin-1 may partially compensate for the side effects caused by systemic VEGF inhibition due to its VEGF-like physiological properties, it is important to investigate how other angiogenic mediators such as netrin-1 respond to systemic VEGF inhibition.

In studies investigating the physiological processes mediated by netrin-1, functional roles of the molecule have been reported that have involved increased NO synthesis [21], enhanced angiogenesis [22], correction of impaired renal functions due to ischemia [23], protective and anti-inflammatory effects on the vascular endothelium [24], and the maintenance of the integrity of the blood-brain barrier [25].

Netrin-1 has been shown to increase the progression of diabetic retinopathy due to its pro-angiogenic effects [4]. Liu et al. reported higher netrin-1 and VEGF concentrations in the vitreous humor of PDR patients [26]. In another study, enhanced BRB breakdown was observed in rats after intravitreal injection of netrin-1 (at a dose of 0.1 μg/mL) [5]. It is an interesting paradox that netrin-1 has both proangiogenic and antiangiogenic effects. Wu et al. explained this dual effect as the fact that netrin-1 functions through different receptors, that different netrin-1 concentrations act oppositely, and that dependence receptors’ (DCC and UNC5H, type I transmembrane receptors that interact with netrin-1), survival and apoptosis signals could have a certain effect on angiogenesis. However, they reported that many studies and molecular evidence are needed to fully shed light on this issue [27]. Although this study did not evaluate vitreous and aqueous humor concentrations, the anti-VEGF increase in the vitreous humor may lead to an upregulated expression of netrin-1 that may decrease treatment efficacy. Therefore, according to the results of this study, a drug that could inhibit both VEGF and netrin-1 expression or signaling might be associated with better clinical outcomes.

This study has some limitations. First, the sample size was small and since the systemic side effects of intravitreal anti-VEGF treatment are rare, it was not possible to perform a correlation analysis between patients with and without side effects, and between serum netrin-1 and VEGF levels. Even though serum VEGF levels were decreased significantly in both NPDR and PDR groups after 1 week and 1 month, serum netrin-1 levels were increased significantly in both examinations of the NPDR group but only in the first-month measurements of the PDR group. Hence, the severity of diabetic retinopathy may cause a delayed netrin-1 response. Increased serum netrin-1 response to VEGF decrease may also be delayed as the general health condition of the patients deteriorate.

## Conclusions

Many ophthalmologists avoid the use of intravitreal bevacizumab based on the studies with the hypothesis that low amounts of drug reaching the systemic circulation after intravitreal bevacizumab injection may cause systemic side effects by inhibiting systemic VEGF [9–11]. However, this study revealed that netrin-1, which has VEGF-like activity against systemic VEGF inhibition, increased and induced a compensatory response that prevented the impairment of physiological function. Clinicians should consider the results of this study in their choice of intravitreal anti-VEGF drugs. However, it should be kept in mind that compensatory netrin-1 response may be delayed and insufficient in patients with poor general health. Further studies investigating the netrin-1 response in the vitreous and aqueous humor after anti-VEGF treatments may shed light on new treatment options for DME.

## Data Availability

The datasets used and/or analysed during the current study are available from the corresponding author on reasonable request.

## Abbreviations

AMD: Age-related macular degeneration
BCZ: Bevacizumab
BRB: Blood-retinal barrier
CAT: Cube average thickness
CDVA: Corrected distance visual acuity
DCC: Deleted in colorectal cancer
DME: Diabetic macular edema
DR: Diabetic retinopathy
ELISA: Enzyme-linked immunosorbent assay
ETDRS: Early Treatment Diabetic Retinopathy Study
HD SD: *High-definition* spectral-domain
HMG-CoA: Hydroxymethylglutaryl-coenzyme A
HT: Hypertension
LogMAR: A logarithm of the minimum angle of resolution
NSAIDs: Non-steroidal anti-inflammatory drugs
NPDR: Non-proliferative diabetic retinopathy
OCT: Optic coherence tomography
PDR: Proliferative diabetic retinopathy
RBZ: Ranibizumab
SPSS: Statistical Package for the Social Sciences
UNC5H: Uncoordinated-5 homolog
VEGF: Vascular endothelial growth factor
